# Pregnancy-associated triple-negative breast cancer: A case report and literature review

**DOI:** 10.1097/MD.0000000000040059

**Published:** 2024-10-11

**Authors:** Weichao Bao, Xiaolin Ma, Yuan Xue, Xin Zou, Ying Guo

**Affiliations:** a School of Clinical Medicine, Shandong Second Medical University, Weifang, Shandong Province, China; b Department of Oncology, Affiliated Hospital of Shandong Second Medical University, Weifang, Shandong Province, China; c Department of Endocrinology and Metabolic Diseases, Affiliated Hospital of Shandong Second Medical University, Weifang, Shandong Province, China.

**Keywords:** case report, PABC, systematic treatment, TNBC

## Abstract

**Rationale:**

The incidence of pregnancy-associated breast cancer (PABC) is relatively low, but it has been increasing in recent years. The onset of PABC causes serious harm to the fetus and the mother due to the unique physiological characteristics of pregnancy, which poses a particular challenge to clinicians. This article reports a case of pregnancy-associated triple-negative breast cancer and describes the patient characteristics and systematic treatment of this type of breast cancer.

**Patient concerns:**

A 33-year-old woman was admitted to hospital with a left breast mass that had appeared more than a year earlier. She was a second-time pregnant woman with a single live intrauterine fetus at 23 + 4 weeks of gestation. During the examination of the left breast, a 6 by 8 cm sized mass can be observed on the upper outer quadrant.

**Diagnosis:**

Pregnancy-associated triple-negative breast cancer.

**Intervention:**

The patient underwent a breast ultrasound which showed a left breast mass and the diagnosis was confirmed by a puncture biopsy of the left breast mass. The pregnancy was terminated after multidisciplinary discussion, taking into account the wishes of the patient and her family. After termination of the pregnancy, all treatments were given according to the standard triple-negative breast cancer (TNBC) treatment protocol. The patient was treated with neoadjuvant chemotherapy with epirubicin in combination with docetaxel (TE) in cycles of 21 days. After 3 cycles of TE, a modified radical mastectomy for left breast cancer was performed, and the appropriate radiotherapy and chemotherapy treatments were carried out in sequence.

**Outcomes:**

After the surgery, the disease-free survival for the patient was 3 months until local metastases were diagnosed. Thus the radiotherapy and chemotherapy were carried out, and then the patient was in good general condition with no recurrence or metastases.

**Lessons:**

Clinicians need more research into the diagnosis, treatment and prognosis of PABC. Improving the rate of early diagnosis and using standardized and individualized comprehensive treatment plans will minimize fetal damage and improve survival and quality of life for patients.

## 1. Introduction

Breast cancer is one of the most common invasive cancers in women and is the second cancer frequently occurring worldwide of newly-diagnosed cancers.^[1,2]^ Pregnancy-associated breast cancer (PABC) is defined as newly diagnosed breast cancer during pregnancy or within 1 year of delivery.^[3]^ Although PABC is relatively rare, its incidence has increased in recent years. The impact of systemic therapy on the fetus presents a particular challenge to clinicians. Compared with common breast cancer, PABC is characterized by a low rate of early detection and poor prognosis. PABC is usually diagnosed in the middle to late stages,^[4]^ of which the onset can be extremely detrimental to the fetus and the mother. In order to detect and diagnose PABC in time and standardize the treatment, we retrospectively analyzed 1 case of PABC admitted to our hospital, and the case report is as follows.

## 2. Case presentation

**Figure 1.**
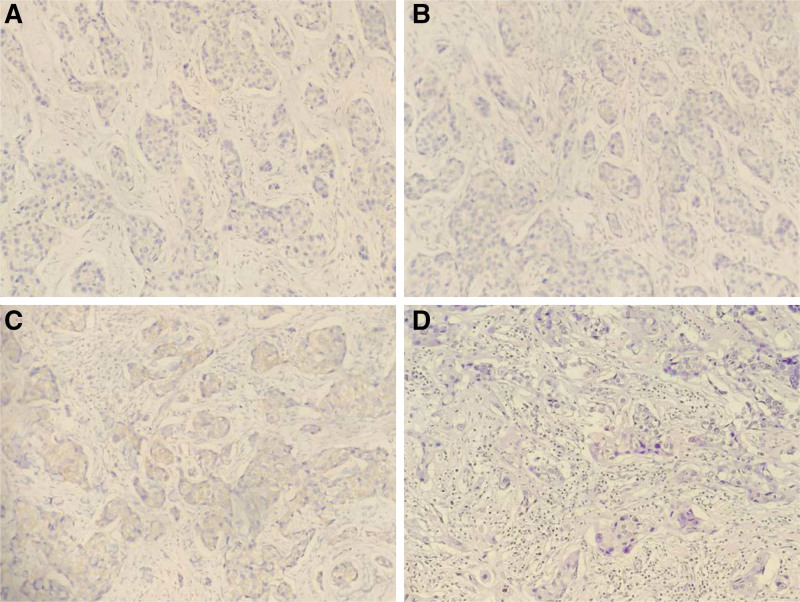
Puncture biopsy pathological results of the left breast mass. (A–C) Original magnification × 200: The tumor cells were negative for ER, PR and HER2 in the breast mass. (D) Original magnification × 200: HE staining showed invasive ductal carcinoma in the left breast mass. ER = estrogen receptor, HER2 = human epidermal receptor-2, PR = progesterone receptor.

The patient was a 34-year-old woman who had a full-term delivery of her first child. In 2015, the patient inadvertently found a jujube-like mass in her left breast without pain, fever, nipple discharge, and was not concerned. In March 2016, during her second child’s fifth month of pregnancy, the left breast mass gradually increased in size to approximately 6 × 4 cm, with redness and swelling and localized skin breakdown in the outer upper quadrant. As for family history, the mother of the patient had a long history of cervical cancer. Breast ultrasound showed a hypoechoic area of approximately 6.8 × 4.3 cm above the left nipple, and bilateral enlarged axillary lymph nodes. On March 23,2016, the patient went to the Affiliated Hospital of Weifang Medical University for a puncture biopsy of the left breast mass, and the pathology showed invasive ductal carcinoma. Immunohistochemistry evaluation showed that the staining was ER-negative and PR-negative, and human epidermal receptor-2 (HER2) staining was also negative as shown in Figure 1. The final diagnosis was invasive ductal carcinoma of the left breast associated with pregnancy.

**Figure 2.**
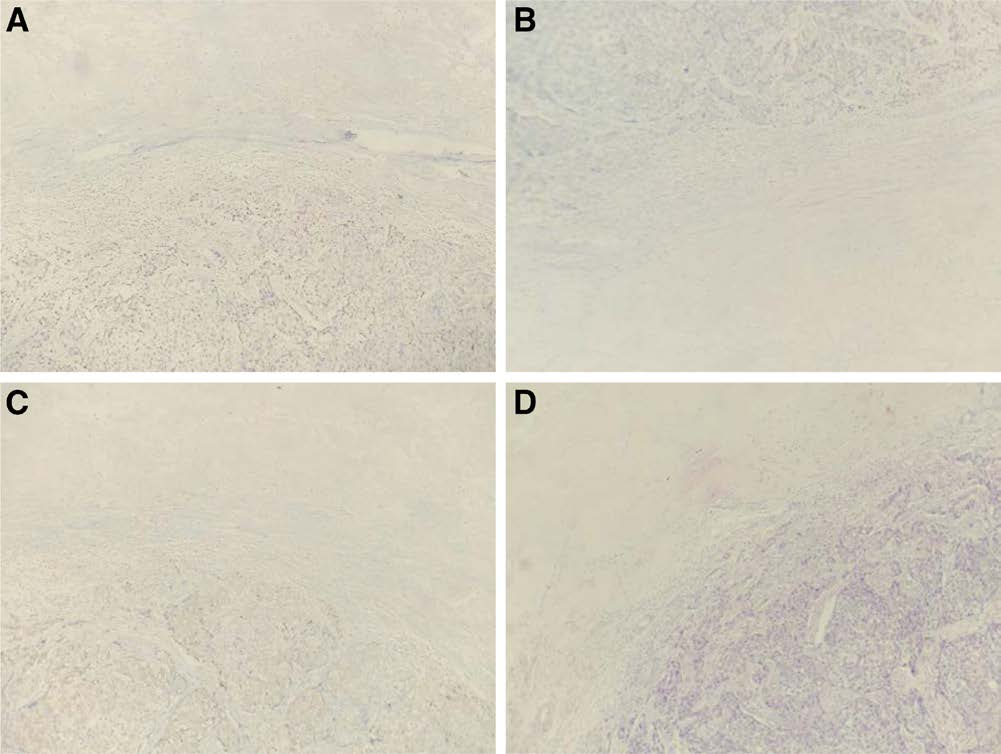
Postoperative pathological results of the left breast mass. (A–C) Original magnification × 100: the tumor cells were negative for ER, PR and HER2 in the breast mass. (D) Original magnification × 100: HE staining showed invasive ductal carcinoma in the left breast mass with necrosis. ER = estrogen receptor, HER2 = HER2 = human epidermal receptor-2, PR = progesterone receptor.

The patient decided to terminate her pregnancy after a multidisciplinary discussion, taking into account her condition and the wishes of her family. A transabdominal injection of ethacridine lactate combined with mifepristone was performed on March 31, 2016 to abort a stillborn male fetus. After the condition of the patient was stabilized, she continued her treatment in the Thyroid and Breast Surgery Department of our hospital. After a comprehensive evaluation by a multidisciplinary team of physicians, together with the medical history and examination of the patient, 3 cycles of preoperative neoadjuvant chemotherapy were given in April 2016, specifically the TE regimen: epirubicin 120 mg d1 and docetaxel 100 mg d2 with 21 days as a cycle. A modified radical mastectomy for left breast cancer was performed under general anesthesia on June 3, 2016. Post-operative pathology revealed lymphocyte-rich invasive ductal carcinoma grade III with necrosis in the left breast, localized skin ulceration and no base or nipple carcinoma, and 21 axillary lymph nodes with no metastatic cancer. Immunohistochemistry evaluation showed that the staining was ER-negative and PR-negative, and HER2 staining was also negative as shown in Figure 2. No cancerous emboli were detected in the CD34 and D2-40 vasculature.

**Figure 3.**
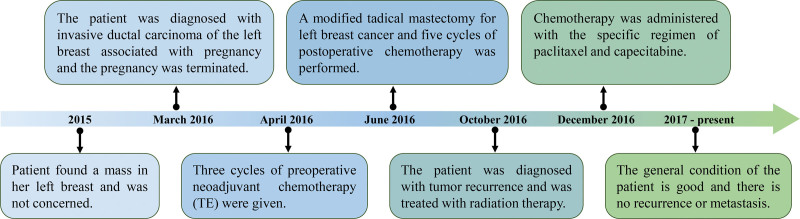
The important milestones related to diagnoses and interventions.

The regimen for the 5 cycles of postoperative chemotherapy was docetaxel 120 mg d1 + pirarubicin 50 mg d1 and 30 mg d2 for 21 days. Chemotherapy was completed in September 2016. On October 3, 2016, the patient was presented to our hospital for treatment, since there was a nodule on the left side of the surgical scar, which had gradually increased in size after surgery, accompanied by multiple nodules under the skin on the left side of the chest wall near the axilla. A breast ultrasound was performed, which showed hypoechoic area about 8.7 × 3.4 mm in the left breast and axilla. The patient was diagnosed with tumor recurrence and was treated with radiation therapy at a prescribed dose of 50 Gy (2 Gy per session, 25 sessions) and 10 sessions of field reduction of the left-sided chest mass. On November 18, 2016, radiation therapy was completed. On December 21, 2016, the patient underwent breast and superficial lymph node ultrasound, which showed 0.6 × 0.4 cm subcutaneous hypoechoic area in the left breast area and negative cervical and glenoid fossa lymph nodes. Three cycles Chemotherapy was administered, with the specific regimen of paclitaxel 180 mg d1 + capecitabine 1.5g bid d1-14, with 21 days as a cycle. The adherence and tolerability of the patient was good throughout the course of treatment. After systematic treatment, the patient was followed up on time and to date, the patient’s general condition is good and there is no recurrence or metastasis. The important milestones related to diagnoses and interventions is shown in Figure 3.

## 3. Discussion

Some studies have reported that the incidence of PABC is approximately 0.2% to 2.5% of all breast cancers.^[5,6]^ Furthermore, it accounts for 6% to 15% of breast cancer patients under 40 years old that associated with pregnancy.^[7,8]^ Breast cancer is a highly heterogeneous group of malignancies at the molecular level,^[9]^ with wide variations in histological morphology, immunophenotype and biological behavior. Triple-negative breast cancer is 1 of the more specialized subtypes with the following biological characteristics. The incidence is higher in certain races and in women with breast cancer susceptibility gene-1 mutations.^[10]^ The tumor has a large volume, and its pathological type is often high-grade invasive ductal carcinoma. Tumor cells mainly express these receptors such as basal-type cytokeratins, P-calmodulin, epidermal growth factor receptor and p53, androgen receptor, E-calmodulin and cyclin D. Compared with conventional breast cancer, the disease-free survival and overall survival were significantly shorter, but the incidence of visceral metastases was higher than that of bone metastases.^[11–14]^

Amant et al state that therapeutic abortion is not routinely recommended and that breast cancer treatment can be carried out in conjunction with continued pregnancy. Human chorionic gonadotropin induces cell differentiation, apoptosis and growth inhibition in mammary epithelial cells and breast cancer cells. A full-term pregnancy leads to complete cell differentiation, and a sudden drop in hormones due to artificial abortion may make the cells more vulnerable.^[15]^ Thus, termination of pregnancy does not improve the patient’s prognosis, which conversely has its drawbacks.^[16,17]^ However, for patients with advanced tumors and poor prognosis in early pregnancy, abortion may be considered to avoid delaying diagnosis and treatment.^[16]^ Treatment of PABC patients should be based on the standard of care for common breast cancers. Meanwhile, it is necessary to combine with the tumor type, gestation period, tumor stage and patient preference.^[15]^ Compared with common breast cancers, the proportion of PR-negative and triple-negative breast cancer (TNBC) are significantly higher in PABC. Especially, the TNBC is accounted for almost a third of the total. According to the analysis of the immunohistochemical results, the patient was diagnosed with pregnancy-associated TNBC and a large tumor burden with local skin ulcer. Considering the adverse impact of neoadjuvant chemotherapy and subsequent treatments on the fetus, the patient ultimately decided to terminate the pregnancy and undergo standardized treatment.

In the field of conventional chemotherapy, standard anthracycline and paclitaxel-based chemotherapy regimens are used throughout the treatment of TNBC. According to the 2015 China Anti Cancer Association guidelines, the A(E)T and TAC regimens are recommended. Furthermore, the total number of cycles of neoadjuvant chemotherapy and adjuvant chemotherapy is generally 6 to 8. Eventually, the ET (epirubicin + docetaxel) chemotherapy regimen was used for the patient. After 3 cycles of neoadjuvant chemotherapy, the tumor shrank and surgery was performed, followed by 5 cycles of postoperative chemotherapy. The postoperative chemotherapy regimen was the same as the preoperative chemotherapy regimen. Taking into account the phenomenon of possible drug resistance and side effects in patients, different drugs from the same regimen were chosen to replace the chemotherapy.

It is an indication for postoperative radiotherapy of breast cancer that the number of axillary lymph node metastasis up to 4 or more. The chest wall radiotherapy should be added postoperatively, especially for the primary tumor >5 cm with fixation, adhesions, and invasion of the chest wall and skin.^[18]^ One month after modified radical mastectomy with chemotherapy, the local recurrence of the tumor was diagnosed. In conjunction with the opinion of the radiotherapy department, 50 Gy of radiotherapy (2 Gy per session, 25 sessions) and 10 sessions of shrinking field were given. According to whether receiving postoperative radiotherapy or not, Yao et al^[19]^ grouped 22,802 female TNBC patients in the SEER database. It was demonstrated by COX regression analysis that postoperative radiotherapy resulted in a significant survival benefit for TNBC patients.

Compared with chemotherapy and neoadjuvant therapy, radiotherapy for TNBC has a good effect only in local treatment. However, breast cancer is essentially a systemic and heterogeneous disease. Not only the local irradiation is necessary, but also the systemic control of the disease cannot be neglected.^[18]^ The advanced TNBC was categorized into paclitaxel-sensitive and paclitaxel-failure types by the 2022 Chinese Society of Clinical Oncology Breast Cancer Annual Meeting. As for paclitaxel-sensitive type, the highest priority recommendation is single agent paclitaxel or combination therapy. The former include albumin-paclitaxel, docetaxel and paclitaxel, while the latter include TX (paclitaxel + capecitabine) and GT (gemcitabine + paclitaxel) and TP (paclitaxel + cisplatin) regimens. Therefore, considering the patient’s sensitivity to paclitaxel analogs, the TX (paclitaxel + capecitabine) regimen was used after the radiotherapy was completed, based on the relevant 2016 guidelines. At the end of 3 cycles of treatment, the disease of the patient was stabilized. There has been no recurrence of the disease since follow-up.

It is an emerging area of research that using platinum-containing chemotherapy regimens in patients with TNBC. A meta-analysis showed that the pathological complete response of TNBC patients can be significantly improved by the intensive neoadjuvant chemotherapy with platinum, with the rate of hematological adverse events was lower.^[20]^ As for targeted therapy, the poly ADP ribose polymerase inhibitor Olaparib fills gap in the precision treatment of TNBC. In 2018, the Olaparib was approved by FDA for the treatment of patients with deleterious or suspected deleterious germline BRCA-mutated, HER2-negative metastatic breast cancer who have been treated with chemotherapy either in the neoadjuvant, adjuvant, or metastatic setting.^[21]^ There are additional treatment options for patients with relapsed metastatic advanced TNBC, provided by the trophoblast cell surface antigen 2 drug Sacituzumab govitecan and chemotherapeutic agents such as Eribulin and Utidelone.^[22]^ In terms of immunotherapy, the programmed cell death protein 1 inhibitor Pembrolizumab has shown breakthrough results in the first-line treatment of programmed death-ligand l-positive advanced TNBC and the neoadjuvant treatment of early TNBC.^[23]^ If the patient has a recurrence of the tumor, the platinum-based drugs can be considered in combination with chemotherapy, and the targeted therapy and immunotherapy can be tried to provide new solutions for the treatment.

It is suggested by the National Comprehensive Cancer Network guidelines that repregnancy does not affect a patient’s prognosis. However, the recurrent metastases in breast cancer often occur within 2 years. Meanwhile, the adverse impacts of anti-tumor drugs on pregnancy should be avoided. Therefore, patients are recommended to pregnancy if there is no recurrent metastasis after 2 years of stopping anti-tumor drugs. In contrast, it is advised to avoid pregnancy for patients with advanced breast cancer.^[24]^ The prognosis for PABC patients is relatively poor, with data showing a 5-year survival rate of 52.1% and a disease-free survival rate of 43.9%, compared with 5-year and 10-year survival rates of 80.0% and 68.6% for conventional breast cancers.^[25]^ The age at onset has been identified as an important factor influencing prognosis, with younger patients having poorer survival.^[26]^ Termination of pregnancy was previously thought to improve prognosis,^[27]^ but some studies have shown that termination does not confer a survival benefit and may even lead to a poorer prognosis.^[28]^

## 4. Conclusions

This article reports a case of pregnancy-associated triple-negative breast cancer that benefited from neoadjuvant chemotherapy, and shrinking the size of the tumor realized the possibility of surgery. Both local and systemic treatments had been considered, and favorable outcomes were achieved. It is regrettable that the patient opted to terminate the pregnancy, as the tumor was too large to be treated directly by surgery, given the irreversible impacts on the fetus. Therefore, more in-depth research is needed by clinicians in the areas of diagnosis, treatment and prognosis for PABC. Improving the rate of early diagnosis and using standardized and individualized comprehensive treatment plans will minimize fetal damage and improve survival and quality of life for patients.

## Author contributions

**Conceptualization:** Weichao Bao, Xiaolin Ma, Yuan Xue.

**Data curation:** Weichao Bao.

**Formal analysis:** Weichao Bao.

**Funding acquisition:** Xiaolin Ma, Ying Guo.

**Investigation:** Weichao Bao, Xiaolin Ma.

**Methodology:** Weichao Bao, Xiaolin Ma.

**Project administration:** Weichao Bao, Xin Zou.

**Resources:** Xiaolin Ma, Ying Guo.

**Software:** Weichao Bao, Ying Guo.

**Supervision:** Xiaolin Ma, Ying Guo.

**Validation:** Xiaolin Ma, Yuan Xue, Xin Zou.

**Visualization:** Weichao Bao, Yuan Xue.

**Writing – original draft:** Weichao Bao, Xiaolin Ma.

**Writing – review & editing:** Ying Guo.
